# Anti-oxidant inhibition of hyaluronan fragment-induced inflammatory gene expression

**DOI:** 10.1186/1476-9255-5-20

**Published:** 2008-11-05

**Authors:** Michael Eberlein, Kara A Scheibner, Katharine E Black, Samuel L Collins, Yee Chan-Li, Jonathan D Powell, Maureen R Horton

**Affiliations:** 1 Department of Medicine, Johns Hopkins Bayview Medical Center, Baltimore, MD, USA; 2 Department of Medicine, Johns Hopkins University School of Medicine, Baltimore, MD, USA; 3 Department of Oncology, Johns Hopkins University School of Medicine, 1830 E. Monument street, 5^th ^floor, Baltimore, MD, USA

## Abstract

**Background:**

The balance between reactive oxygen species (ROS) and endogenous anti-oxidants is important in maintaining healthy tissues. Excessive ROS states occur in diseases such as ARDS and Idiopathic Pulmonary Fibrosis. Redox imbalance breaks down the extracellular matrix component hyaluronan (HA) into fragments that activate innate immune responses and perpetuate tissue injury. HA fragments, via a TLR and NF-κB pathway, induce inflammatory gene expression in macrophages and epithelial cells. NAC and DMSO are potent anti-oxidants which may help balance excess ROS states.

**Methods:**

We evaluated the effect of H_2_O_2_, NAC and DMSO on HA fragment induced inflammatory gene expression in alveolar macrophages and epithelial cells.

**Results:**

NAC and DMSO inhibit HA fragment-induced expression of TNF-α and KC protein in alveolar and peritoneal macrophages. NAC and DMSO also show a dose dependent inhibition of IP-10 protein expression, but not IL-8 protein, in alveolar epithelial cells. In addition, H_2_O_2 _synergizes with HA fragments to induce inflammatory genes, which are inhibited by NAC. Mechanistically, NAC and DMSO inhibit HA induced gene expression by inhibiting NF-κB activation, but NAC had no influence on HA-fragment-AP-1 mediated gene expression.

**Conclusion:**

ROS play a central role in a pathophysiologic "vicious cycle" of inflammation: tissue injury generates ROS, which fragment the extracellular matrix HA, which in turn synergize with ROS to activate the innate immune system and further promote ROS, HA fragment generation, inflammation, tissue injury and ultimately fibrosis. The anti-oxidants NAC and DMSO, by inhibiting the HA induced inflammatory gene expression, may help re-balance excessive ROS induced inflammation.

## Background

In normal physiological conditions a homeostatic balance exists between the formation of reactive oxygen species (ROS) and their removal by endogenous antioxidant scavenging compounds [1]. ROS are commonly produced during inflammatory processes and play an important role in host defense against infections. ROS are also involved in signal transduction and gene activation, and can contribute to host cell and organ damage [2]. When cellular production of ROS overwhelms its antioxidant capacity, a state of oxidative stress is reached leading to serious cellular injuries and contributing to the pathogenesis of several diseases like ARDS, Asthma, COPD, cancer and Idiopathic Pulmonary Fibrosis [3]. The redox imbalance is evident in the interstitial disease Idiopathic Pulmonary Fibrosis in which median time to death after diagnosis is 3 years [4,5]. However, recent reports suggest that counteracting this state of oxidative stress with the anti-oxidant N-acetylcysteine slows down the progression of this deadly disease [6].

Redox imbalance also leads to the breakdown of the extracellular matrix [7-9]. In lung inflammation and fibrosis, the extracellular matrix is not only a target of destruction but also plays an important role in perpetuating and augmenting tissue injury and inflammation via induction of cytokines, chemokines and modulatory enzymes. Hyaluronan (HA), a negatively charged normally high molecular weight glycosaminoglycan, is ubiquitously distributed in the extracellular matrix [10,11]. It is found in the basement membrane of normal lungs, joints and vitreous fluid and makes up about 70% of the glycosaminoglycan content of the lung [12]. It is primarily produced by fibroblasts and to a lesser degree by smooth muscle cells and appears to function in water homeostasis, plasma protein distribution and transportation, joint lubrication, and matrix structure [10,11]. *In vivo*, at sites of inflammation, the high molecular weight HA (size 1 × 10^6 ^KDa) can be depolymerized to lower molecular weight (size 2 × 10^5 ^KDa) fragments via oxygen radicals and enzymatic degradation by hyaluronidase, β-glucuronidase and hexosaminidase [8,9,13]. Furthermore, increased concentrations of HA have been found in bronchoalveolar fluid from patients with sarcoidosis, idiopathic pulmonary fibrosis as well as in the joints of patients with rheumatoid arthritis [14-16]. Recently, HA fragments have been demonstrated to play a significant role in the development of lung inflammation and fibrosis in the bleomycin model of lung injury [17,18].

Upon a noxious insult such as an infection, ischemia, or environmental toxin, the generation of reactive oxygen species (ROS) and hyaluronidases act to break down the extracellular matrix component HA into lower molecular weight fragments [8,9,13]. HA fragments themselves act as endogenous danger signals and activate innate immune responses similar to microbial antigens [19]. Via the Toll like Receptor-2 (TLR-2) pathway, these matrix fragments induce inflammatory gene expression in alveolar macrophages and airway epithelial cells [19]. HA fragments induce the expression of a wide array of inflammatory genes in such as MIP-1α, MIP-1β, RANTES, MCP, IL-8, IP-10, IL-12, MIG, KC, TNF-α, metalloproteases and nitric oxide synthase [20-25].

The potent antioxidants N-acetylcysteine (NAC) and dimethyl sulfoxide (DMSO) have been shown to possess both anti-inflammatory and anti-fibrotic properties [26,27]. They have been shown to inhibit the progression of lung fibrosis in both humans and animal models [6,27-31]. NAC contains a sulfhydryl (SH) group and is metabolized rapidly to L-cysteine, which is a direct precursor to the intracellular antioxidant glutathione (GSH). In addition to restoring the intracellular GSH pool and being a source of intra- and extracellular SH groups, NAC can also act as a direct scavenger of free radicals such as OH and H_2_O_2_. NAC has been shown to have influence on redox-sensitive cell-signaling and transcription pathways, such as NF-κB dependent gene expression [26]. DMSO, a hydroxy radical scavenger, is believed to induce its inhibitory effects via suppression of NF-κB activation [32].

In this report we demonstrate the ability of the antioxidants NAC and DMSO to inhibit HA fragment induced inflammatory gene expression. Furthermore, we demonstrate that ROS synergize with HA fragments to induce inflammatory gene expression. Since ROS are involved in both the generation of HA fragments and enhancing HA fragment induced inflammatory genes, we propose a crucial role for ROS in mediating the inflammatory properties of HA fragments. As HA fragments have been implicated in promoting lung inflammation and fibrosis, the importance of ROS in the generation of and subsequent inflammation induced by HA fragments highlights a potentially important pathway and therapeutic target for inflammatory and fibrotic lung diseases.

## Methods

### Cells

The mouse alveolar macrophage cell line MH-S was purchased from American Type Culture Collection, Rockville, MD. NCI-H292 airway epithelium-like cells (derived from a human pulmonary mucoepidermoid carcinoma) were obtained from the American Type Culture Collection and have been shown to be LPS hyporesponsive [33]. Thioglycollate-elicited peritoneal macrophages were lavaged from female C_3_H/HeJ LPS hyporesponsive mice 4 days after injection of 2 ml of sterile thioglycollate (Sigma-Aldrich). All animal experiments were approved by the Johns Hopkins Committee on Animal Use, and experiments were conducted in accordance to their guidelines and regulations. To exclude effects of contaminating LPS on experimental conditions, cell stimulation was carried out in the presence of polymixin B 10 μg/ml (Calbiochem Novabiochem, La Jolla, CA). We assessed effects of NAC and DMSO on cell viability by trypan blue exclusion as well as by FACS for annexin.

### Chemicals and reagents

Purified HA fragments from human umbilical cords (Calbiochem Novabiochem) are free of protein and other glycosaminoglycans with a peak molecular weight of 200,000 D. The HA fragments still retained their ability to signal despite treatment with proteinase K, DNAse and heat inactivation that effectively removes protein, DNA and heat labile LPS contaminants (data not shown). However, after full digestion with hyaluronidase, the fragments no longer induce gene expression, further negating the effects of possible contaminants. DMSO, H_2_O_2 _and N-acetylcysteine were obtained from Sigma.

### ELISA for protein secretion

ELISA for TNF-α, KC, IL-8 and IP-10 were performed per manufacturer's guidelines (R & D, Minneapolis, MN). Colorimetric changes were measured in an ELISA plate reader and analyzed with Microplate Manager III (BioRad) software.

### Transient Transfections

Transient transfections were performed using lipofectamine 2000 (Invitrogen) or Fugene 6 per manufacturer's guidelines. P-NIFTY NF-κB luciferase reporter construct was purchased from Invivogen and the AP-1 reporter was a kind gift Dr Sekar Reddy. Luciferase expression was measured with a Dual Luciferase Kit (Promega) and a Zylux femtomaster FB-12 luminometer.

### Statistics

Each condition was performed in triplicate for each experiment, and the data presented represent the average of three or more unique experiments. Differences between groups were analyzed using ANOVA with Fisher's PLSD test for pair-wise comparisons (Graphpad). A p-value < 0.05 was considered significant.

## Results

### DMSO inhibits HA fragment induced TNF-α and KC expression macrophages

**Figure 1:**
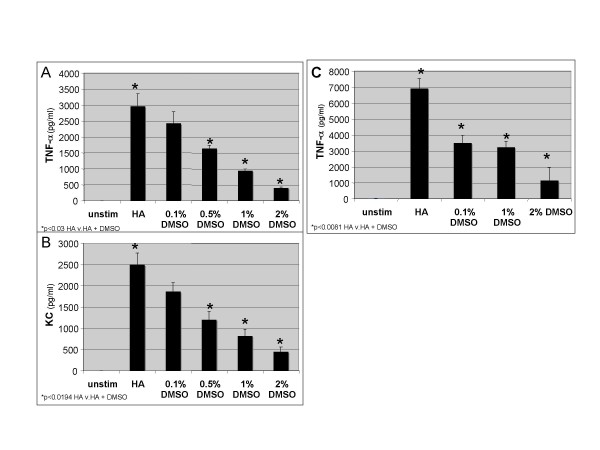
**DMSO inhibits HA fragment induced TNF-α and KC expression macrophages**. ELISA for TNF-α and KC of cultured cell supernatants from **(A, B) **MH-S cells stimulated with HA fragments (250 μg/ml) in the presence of DMSO at 37°C for 18 h. **(C) **PECs were stimulated with HA fragments (250 μg/ml) in the presence of DMSO in concentrations as indicated at 37°C for 18 h and TNF-α was measured in cell supernatants. These data represent the average of 3 identical, independent experiments.

DMSO is a common solvent used to dissolve many biological substances and we have used diluted DMSO alone as a vehicle control. DMSO is also a powerful anti-oxidant that has been reported to decrease LPS induced gene expression [34]. Thus, we wanted to determine the effect of DMSO on HA fragment-induced gene expression. We stimulated the alveolar macrophage cell line MH-S with HA fragments and DMSO for 18 h and cell supernatants were harvested and analyzed for specific chemokine and cytokine expression by ELISA. DMSO markedly inhibits HA fragment induced TNF-α by up to 87% (p-value < 0.03) and inhibits KC by up to 83% (p =< 0.0194) (Figure 1A &1B). To demonstrate that the ability of DMSO to inhibit HA-fragment induced cytokine expression was not idiosyncratic to the MH-S alveolar macrophage cell line, we examined its effect on HA fragment-stimulated genes in primary macrophages. Thioglycollate elicited peritoneal macrophages (PEC) from C3H/Hej LPS hyporesponsive mice were stimulated with HA fragments in the presence of different concentrations of DMSO for 18 h and TNF-α was measured in cell supernatants. DMSO significantly inhibited HA fragment induced TNF-α in primary macrophages in a dose dependent fashion between 49–83%, p = 0.0081(Figure 1C). The fact that these C3H/Hej primary macrophages are LPS resistant further supports the specificity of the HA fragments to induce the inflammatory genes and thus, the inhibitory effect of DMSO. The inhibition of HA fragment induced genes by DMSO was not due to increased cell death as there was equal survival of cells per trypan blue exclusion and FACS for annexin (data not shown). Thus, DMSO inhibits HA fragment induced inflammatory gene expression.

### NAC inhibits HA fragment induced gene expression in macrophages

**Figure 2:**
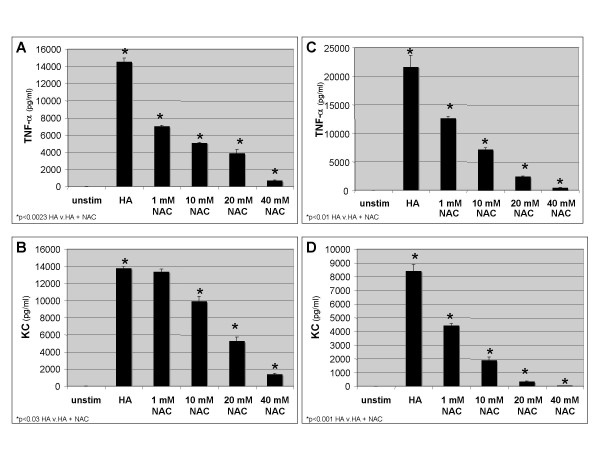
**NAC inhibits HA fragment-induced Gene expression in macrophages**. ELISA for TNF-α and KC of cultured cell supernatants from MH-S (**A, B**) or PEC (**C, D**) cells stimulated with HA fragments (250 μg/ml) in the presence of NAC in concentrations as indicated at 37°C for 18 h. These data represent the average of 3 identical, independent experiments.

Next we wanted to determine if the effect of DMSO was due to its antioxidant properties. Thus, we stimulated macrophages with HA fragments in the presence of the anti-oxidant N-acetylcysteine (NAC), a drug demonstrated to inhibit the progression of lung fibrosis [6]. MH-S macrophages were stimulated with HA fragments and NAC for 18 h and cell supernatants were harvested and analyzed for specific chemokine and cytokine expression by ELISA. We demonstrated that NAC markedly inhibits HA fragment-induced TNF-α by up to 96% (p < 0.0023) and inhibits KC by 29–83% (p < 0.03) (Figure 2A &2B). Furthermore, NAC also inhibited HA fragment-induced genes in primary peritoneal macrophages (PEC). PECs were stimulated with HA fragments and NAC for 18 h and TNF-α and KC were measured in cell supernatants. NAC significantly inhibited HA fragment induced TNF-α (42–98%, p < 0.01) and KC (48–99%, p = 0.001) in a dose dependent fashion (Figure 1C &1D). The inhibition of HA fragment induced genes by NAC was not due to increased cell death as there was equal survival of cells per trypan blue exclusion and FACS for annexin (data not shown). Thus, the antioxidant NAC significantly inhibits HA fragment-induced inflammatory gene expression.

### NAC inhibits HA fragment-induced gene expression in airway epithelial cells

**Figure 3:**
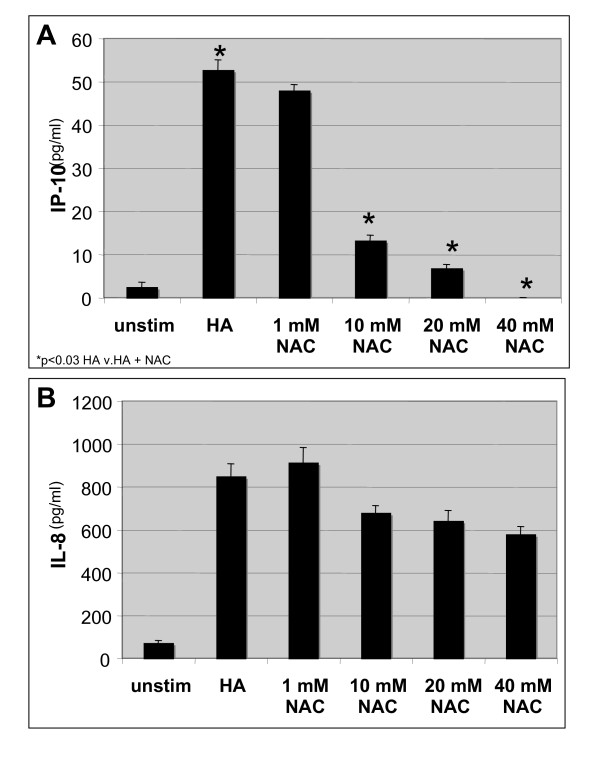
**NAC inhibits HA fragment-induced gene expression in airway epithelial cells**. ELISA for **(A) **IP-10 and **(B) **IL-8 of cultured cell supernatants from NCI H292 cells stimulated HA fragments (250 μg/ml) in the presence of NAC in concentrations as indicated at 37°C for 18 h. These data represent the average of 4 identical, independent experiments.

As we have demonstrated that HA fragments significantly induce inflammatory gene expression in airway epithelial cells, we wanted to determine if the anti-oxidant NAC could inhibit HA fragment-induced inflammatory gene expression in alveolar epithelial cells. Thus, cells from the alveolar epithelial cell line H292 were stimulated with HA fragments and NAC for 18 h; cell supernatants were harvested and analyzed for specific chemokine and cytokine expression by ELISA. As was the case for macrophages, NAC markedly inhibits HA fragment-induced IP-10 by 75–100% (p < 0.03) but did not inhibit IL-8 (Figure 3A &3B). Interestingly, NAC did not inhibit HA induced IL-8 induction in the airway epithelial cells. These observations serve to demonstrate the relative specificity of the NAC effect. That is, NAC is not generically inhibiting all HA-induced cytokines, further supporting our observations that NAC (and DMSO) do not affect the viability of the HA fragment stimulated cells. Interestingly, we have previously demonstrated that HA fragments induce IP-10 in airway epithelial cells via a NF-κB dependent pathway where as HA fragments induce IL-8 in the same cells via AP-1 mediated pathway [33]. Thus, NAC inhibits HA fragment-induced IP-10 but not IL-8 in the airway epithelial cell line H292.

### ROS synergize with HA fragments to induce inflammatory gene expression

**Figure 4:**
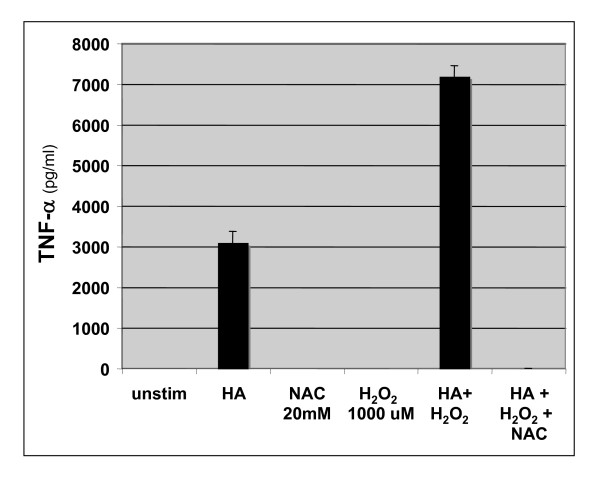
**ROS synergize with HA fragments to induce inflammatory gene expression**. ELISA for TNF-α of cultured cell supernatants from MH-S cells stimulated with HA fragments (250 μg/ml) in the presence of H_2_O_2 _(1000 μM) +/- NAC (20 mM) for 18 h. These data represent the average of 4 identical, independent experiments.

Given that ROS are often found in the inflammatory milieu and that ROS can fragment high molecular weight HA into the inflammatory lower molecular weight fragments, we wanted to evaluate the effect of ROS on HA fragment-induced inflammatory gene expression. MH-S alveolar macrophages were stimulated with HA fragments in the presence of H_2_O_2 _and or NAC. Although HA fragments induced TNF-α protein expression, neither H_2_0_2 _nor NAC alone induced appreciable levels of TNF-α (Figure 4). However, HA fragments synergized with H_2_0_2 _to significantly increase TNF-α expression (Figure 4). Furthermore, NAC was able to inhibit both HA fragment-induced gene expression as well as the synergy between HA and H_2_0_2_. Thus, ROS can synergize with HA fragments to augment inflammatory gene expression by macrophages.

### NAC and DMSO inhibit HA fragment-induced NF-κB activation

**Figure 5:**
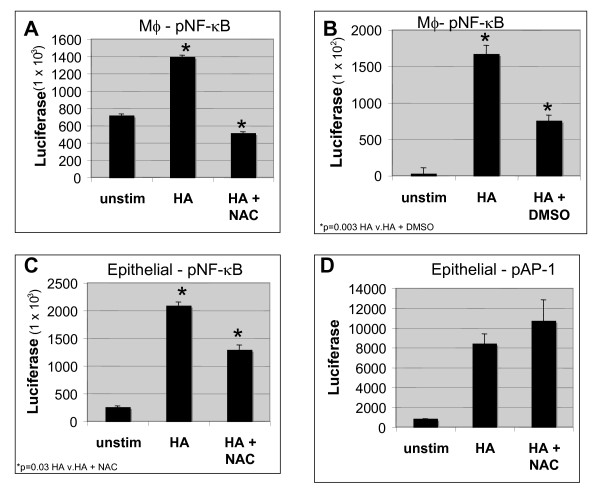
**NAC and DMSO inhibit HA fragment-induced NF-κB, but not AP-1 activation**. MH-S cells were transiently transfected with an NF-κB driven luciferase reporter construct for 18 h prior to stimulation with HA fragments (250 μg/ml) +/- 10 nM NAC **(A) **or DMSO 1% **(B) **for an additional 18 h. Cell lysates were harvested and assayed for luciferase expression and results are displayed as fold induction of luciferase activity. NCI H292 cells were transfected with NF-κB **(C) **or AP-1 **(D) **luciferase reporter constructs for 18 h prior to stimulation with HA fragments (250 μg/ml) +/- 10 nM NAC for an additional 18 h. Promoter activity was assayed by luciferase activity and results are displayed as fold induction of luciferase activity. These data represent the average of 6 identical, independent experiments.

Given the differential inhibition of HA fragment-induced gene expression in airway epithelial cells, we wanted to determine if NAC and DMSO were inhibiting HA fragment induced gene expression via NF-κB. MH-S cells were transiently transfected with an NF-κB driven luciferase reporter construct for 18 h prior to stimulation with HA fragments and NAC for an additional 18 h. Cell lysates were harvested and assayed for luciferase expression. NAC markedly inhibited HA fragment-induced NF-κB activation by 64%, p = 0.0281 (Figure 5A). To determine if DMSO was also inhibiting HA fragment-induced gene expression via a NF-κB dependent pathway, MH-S cells were transiently transfected with an NF-κB driven luciferase reporter construct for 18 h prior to stimulation with HA fragments and DMSO for an additional 18 h. Cell lysates were harvested and assayed for luciferase expression. DMSO markedly inhibited LMW HA-induced NF-κB activation by 55%, p = 0.003 (Figure 5B).

Similarly, H292 airway epithelial cells were transiently transfected with an NF-κB driven luciferase reporter construct for 18 h prior to stimulation with HA fragments and NAC for an additional 18 h. Cell lysates were harvested and assayed for luciferase expression. NAC markedly inhibited HA fragment-induced NF-κB activation by 42%, p = 0.03 (Figure 5C). Since, HA-induced IL-8 expression is AP-1 dependent we wanted to determine the affect of NAC on AP-1 driven reporter constructs. H292 airway epithelial cells were transiently transfected with an AP-1 driven luciferase reporter construct for 18 h prior to stimulation with HA fragments and NAC for an additional 18 h. Cell lysates were harvested and assayed for luciferase expression. NAC did not inhibit HA fragment-induced AP-1 activation consistent with the inability of NAC to inhibit HA fragment-induced IL-8 (Figure 5D). Thus, the antioxidants NAC and DMSO inhibit HA fragment-induced inflammatory via a NF-κB dependent pathway but do not affect HA-induced AP-1.

## Discussion

The microenvironment of injured tissues and cells plays an essential role in determining the extent and intensity of the inflammatory milieu. Although ROS are important in the successful resolution of certain inflammatory states, such as infections, an imbalance in the redox homeostasis can result in excessive tissue injury and cell death [3]. We propose one mechanism by which oxidative stress may augment and perpetuate inflammation is via ROS induced fragmentation of the extracellular matrix [8,9]. In particularly, ROS lead to degradation of the normally protective high molecular weight HA into pro-inflammatory HA fragments [8,9]. These low molecular weight fragments further exacerbate the inflammation via the induction of inflammatory chemokines and cytokines by macrophages and lung epithelial cells. In this manuscript, we demonstrate for the first time synergy between HA fragments and ROS to induce inflammatory gene expression and the inhibition of these genes by anti-oxidants. The anti-oxidants NAC and DMSO strongly inhibit HA fragment induced TNF-α and KC in alveolar macrophages. The effect of anti-oxidants was not limited to phagocytic cells as we demonstrate a similar inhibition of HA fragment induced IP-10 in a human airway epithelial cells. Mechanistically the anti-oxidants NAC and DMSO inhibit the HA fragment activation of NF-κB in these cells. The anti-oxidants NAC and DMSO had no effect on HA fragment induced AP-1 activation and IL-8 expression in alveolar epithelial cells. Thus, the anti-oxidants NAC and DMSO can mitigate effects of the pro-inflammatory HA fragments released at sites of tissues injury and redox imbalance.

We have demonstrated that HA fragments, as an endogenous danger signal, activate an innate immune response via TLR-2 ligation and NF-κB activation in macrophages and epithelial cells [19,33]. However, others have demonstrated that other forms of LMW HA fragments can signal via TLR-4 or both TLR-2 and TLR-4 [17,35,36]. Activated alveolar macrophages are a major source of endogenous ROS and exhibit the most potent capacity to generate ROS compared to macrophages from other anatomic compartments [37]. ROS generation in alveolar macrophages in response to TLR ligation has been reported for the TLR-4 ligand LPS [38]. Ndengle et al demonstrate in alveolar macrophages that ROS enhance the gene transcription of TNF-α in response to LPS [38]. Involvement of ROS in inflammatory gene expression by TLR-4 has been suggested using antioxidants [39]. Pre-treatment of neutrophils with the anti-oxidant NAC or tocopherol prevented LPS-induced production of pro-inflammatory cytokines [39]. The exact molecular source of ROS upon LPS challenge was recently discovered by Park et al who showed that LPS-induced ROS generation is mediated by direct interaction of TLR-4 with NADPH oxidase 4 (Nox4) [40]. LPS-signaling via TLR 4 shares many similarities to HA fragment signaling via TLR-2 [19]. Thus it was important to exclude LPS signaling effects via LPS contamination of LMW HA fragment preparations. To this end, all experiments were performed in the presence of polymyxin B and the primary macrophages were derived from LPS hyporesponsive CeH/HeJ mice. It has been shown in the past that under these conditions LPS fails to induce inflammatory gene up-regulation [21,22,25].

Our data add to the growing body of literature highlighting the role of ROS as important signaling molecules that are able to modulate various gene transcription via activation of redox-sensitive protein kinases and transcription factors [41]. ROS have been identified as second messengers in cells, and play a role in receptor signaling and post-translational modification of signaling molecule activity [41,42]. Many kinases involved in direct or indirect activation of NF-κB are affected by oxidants and therefore, have the potential to alter NF-κB activity [41,42]. The transcription factor NF-κB plays a major role in coordinating innate and adaptive immunity, cellular proliferation, apoptosis and development and is a key transcription factor for many HA fragment induced inflammatory gene expression. In macrophages and alveolar epithelial cells, HA fragments can induce chemokines via activation of the NF-κB pathway [33,43]. We have shown that the anti-oxidants NAC and DMSO inhibit the HA fragment induced inflammatory gene expression by interfering with HA fragment-induced activation of NF-κB. This finding is in line with other investigations showing the importance of ROS in the activation of NF-κB [44]. Schreck et al. were the first to demonstrate that direct addition of ROS, specifically H_2_O_2 _to the culture medium of a subclone of Jurkat cells could activate NF-κB. Now several lines of evidence support a model suggesting that ROS mainly activate NF-κB via IKK activation and IκBa degradation [2,44].

Pulmonary fibrosis is associated with chronic inflammation, increased ROS, and accumulation and turnover of extracellular matrix. During lung inflammation in both human diseases and bleomycin injured animal models, activated phagocytes release large amounts of reactive oxygen species (ROS) that have been demonstrated to be involved in tissue injury and to impede tissue repair, thus leading to pulmonary fibrosis [3,27]. Anti-oxidant treatment protects against bleomycin-induced lung damage in rodents [27-30]. Additionally, mice deficient in extracellular Superoxide Dismutase (SOD) develop an exaggerated fibrosis in response to bleomycin [45]. Inghilleri et al. compared the in situ oxidative burden and anti-oxidant enzyme activity in bleomycin-injured rat lungs and normal controls and found after treatment with bleomycin, ROS production was enhanced in both phagocytes and in type II alveolar epithelial cells [46]. Interestingly Manoury et al. have demonstrated that mice deficient in the p47phox subunit of the NADPH oxidase complex are unable to produce ROS via the NADPH oxidase pathway and do not develop pulmonary fibrosis after intranasal administration of bleomycin [47]. In addition, recent clinical trials show favorable effects on lung function decline in patients with idiopathic pulmonary fibrosis treated with high doses of N-acetylcysteine [6].

## Conclusion

**Figure 6:**
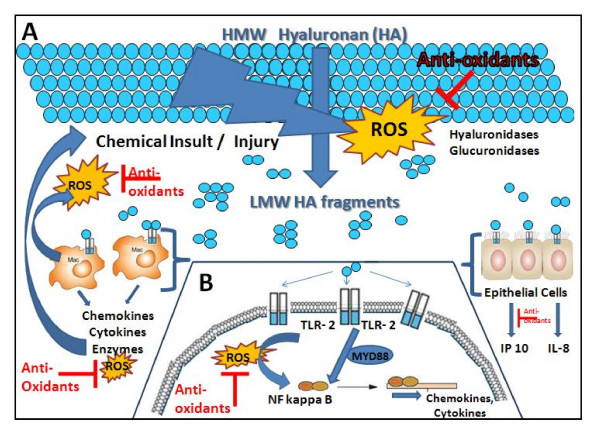
**The central role of ROS in a pathophysiologic "vicious cycle"**: A) Tissue injury generates ROS, which mediate fragmentation of the extracellular matrix HA. B) Fragmented HA and ROS synergize to activate the innate immune system via TLR-2, followed by further production of ROS, activation of NF-κB and expression of inflammatory cytokines and chemokines, promoting further inflammation. This cycle perpetuates continued fragmentation of the extracellular matrix HA and generation of ROS, thus leading to further injury, inflammation and ultimately fibrosis. Anti-oxidants have the potential to ameliorate this vicious cycle.

In conclusion we have shown that the anti-oxidant NAC inhibits HA fragment-induced cytokine expression via NF-κB inhibition in macrophages and epithelial cells. Taken together our data provides further insight into the basic mechanisms of beneficial effects anti-oxidants have demonstrated in animal models of pulmonary fibrosis and possibly in patients with Idiopathic Pulmonary Fibrosis. The findings in this investigation point towards a central role of ROS in the pathophysiologic "vicious cycle" of inflammation: tissue injury generates ROS, which generate fragments of the extracellular matrix HA, which in turn synergize with the ROS to activate the innate immune system via TLR-2. Activation of the immune system leads to further production of ROS by activated macrophages, activation of NF-κB and induction inflammatory cytokines and chemokines that promote further inflammation and continued fragmentation of the extracellular matrix HA, generation of ROS, more injury, more inflammation and ultimately fibrosis (Figure 6). Thus, multi-targeted therapeutic interventions addressing this self perpetuating spiral of tissue injury, ROS production, matrix degradation that leads to further matrix-induced inflammation may hold a promise of improving clinical outcomes in patients with inflammatory diseases in the future.

## Competing interests

The authors declare that they have competing interests.

## Authors' contributions

ME co-conceived the study, carried out the NAC and DMSO cellular studies, participated in the ELISAs and transfections and drafted the manuscript. KAS participated in the ELISAs and transfections. KEB participated in the ELISAs and transfections. SLC preformed the FACS experiments. YC participated in the ELISAs, transfections and ROS cellular experiments. JDP participated in the design of the study and helped to draft the manuscript. MRH co-conceived of the study, participated in its design and coordination, performed the statistical analysis and helped draft the manuscript. All authors read and approved the final manuscript.
